# Supplementary material for: Examining the liver–pancreas crosstalk reveals a role for the molybdenum cofactor in β-cell regeneration
Source: Life Sci Alliance. 2024 Aug 19;7(11):e202402771. doi: 10.26508/lsa.202402771 (PMC11333758; doi:10.26508/lsa.202402771)
Supplement: Supplementary file 3 [file LSA-2024-02771_TableS1.docx]

**Supplementary Table 1:** Primers used for amplification of selected genes for qPCR analysis.

| Gene | Forward primer | Reverse primer |
| --- | --- | --- |
| *eef1a1l1* | 5’ GTGCTGTGCTGATTGTTGCT 3’ | 5’ TGTATGCGCT  GACTTCCTTG 3’ |
| *actb1* | 5’ CGAGCAGGAGATGGGAACC 3’ | 5’ CAACGGAAACGCTCATTGC 3’ |
| *mocs1* | 5‘ ACCACTAATTCGCCCAGACA 3‘ | 5‘ CCCATTTGTGGTGACTGCAA 3‘ |
| *long isoform mocs2* | 5’ TTCTGTAACCGAAGCGAGTG 3’ | 5’ AGCTTTGAGTGTGTCGATGC 3’ |
| *short isoform mocs2* | 5’ GCGGAGATTATTACCGTTCC 3’ | 5’ ACAGCCAGAACCACCTTACC 3’ |
| *mocs3* | 5‘ CCTCGTGAATGATGCCTGTG 3‘ | 5‘ TAGTGACGGTCTCAGGAGGA 3‘ |
| *gphna* | 5‘ TCCATGCCCAGATCCACTTT 3‘ | 5‘ TGGAAGGGCGAAGATCAGTT 3‘ |
| *gphnb* | 5‘ AAGTCATAGTGCGGTGGACA 3‘ | 5‘ GCATAAACGTCCTGAGCCAG 3‘ |
| *aox5* | 5‘ CAGCTCTACGACCTGAGGAG 3‘ | 5‘ TCCAGATGCTCCACCACATT 3‘ |
| *aox6* | 5‘ GATTGTCCATGCCTTCCGAC 3‘ | 5‘ TTGTCATTGAGCCAAACCCG 3‘ |
| *suox* | 5‘ GGTCTTTCACAGCTCCTCCT 3‘ | 5‘ ATTTCTGCCCTCCATGACCA 3‘ |
| *xdh* | 5‘ AGCCTGCACTGTAATGGTCT 3‘ | 5‘ TGTACGGGATGGAGTTTGCT 3‘ |
| *g6pc1a* | 5’ TCACAGCGTTGCTTTCAATC 3’ | 5’ AACCCAGAAACATCCACAGC 3’ |
| *pck1* | 5’ AGAACAGCACCATCCTCAGC 3’ | 5’ ACCGTTTTACTCTCCACACG 3’ |
| *pklr* | 5’ AGCTGTTTGAAGAGTTGCGG 3’ | 5’ CGCAGCACTTGTATGAGGAC 3’ |
