# Supplementary material for: Examining the liver–pancreas crosstalk reveals a role for the molybdenum cofactor in β-cell regeneration
Source: Life Sci Alliance. 2024 Aug 19;7(11):e202402771. doi: 10.26508/lsa.202402771 (PMC11333758; doi:10.26508/lsa.202402771)
Supplement: Supplementary file 4 [file LSA-2024-02771_TableS2.docx]

**Tables**

**Supplementary Table 2:** Primers used for amplification of selected genes to clone into the entry vectors of the Gateway system.

| Gene | Forward primer | Reverse primer |
| --- | --- | --- |
| *desi2* | 5’ GGGGACAAGTTTGTACAAAAAAGCAGGCTGCCACCATGGCAAACGAGCCGGTTATCCT 3’ | 5’ GGGGACCACTTTGTACAAGAAAGCTGGGTTCATCGGCGCGGCTGGT 3’ |
| *eno1b* | 5’ GGGGACAAGTTTGTACAAAAAAGCAGGCTGCCACCATGTCTATTCTGAAGATACACG 3’ | 5’ GGGGACCACTTTGTACAAGAAAGCTGGGTTCAGTTCAGTGGATTTCTG 3’ |
| *fynb* | 5’ GGGGACAAGTTTGTACAAAAAAGCAGGCTGCCACCATGGGCTGTGTGCAATGTAA 3’ | 5’ GGGGACCACTTTGTACAAGAAAGCTGGGTCTAGAGGTTGTCTCCGGGC 3’ |
| *gale* | 5’ GGGGACAAGTTTGTACAAAAAAGCAGGCTGCCACCATGCGTCAAAAGATCTTG 3’ | 5’ GGGGACCACTTTGTACAAGAAAGCTGGGTTCAAGGTAGTGTTCCATTACT 3’ |
| *long isoform mocs2* | 5’ GGGGACAAGTTTGTACAAAAAAGCAGGCTGCCACCATGGCTGCCGATGGG 3’ | 5’ GGGGACCACTTTGTACAAGAAAGCTGGGTTCAGCTTTCTTTGGGATGTTTT 3’ |
| *short isoform mocs2* | 5’ GGGGACAAGTTTGTACAAAAAAGCAGGCTGCCACCATGAATACCGAGGTGTCGGT 3’ | 5’ GGGGACCACTTTGTACAAGAAAGCTGGGTTTATCCTCCACTGAGTGGCG 3’ |
| *ndufa4* | 5’ GGGGACAAGTTTGTACAAAAAAGCAGGCTGCCACCATGCTTGCAACCGTGATG 3’ | 5’ GGGGACCACTTTGTACAAGAAAGCTGGGTTTAGAAGTCAGGCCTGTCCTT 3’ |
| *plod3* | 5’ GGGGACAAGTTTGTACAAAAAAGCAGGCTGCCACCATGACTCCGGTGCCCGT 3’ | 5’ GGGGACCACTTTGTACAAGAAAGCTGGGTTCAGGGGTCTACGAATGACACC 3’ |
| *rnf182* | 5’ GGGGACAAGTTTGTACAAAAAAGCAGGCTGCCACCATGGGACAGCTCCCGGA 3’ | 5’ GGGGACCACTTTGTACAAGAAAGCTGGGTTTACGGTGGTACACAGTCCCAAA 3’ |
| *sdf2l1* | 5’ GGGGACAAGTTTGTACAAAAAAGCAGGCTGCCACCATGATGGGGCCGTTAAGAGT 3’ | 5’ GGGGACCACTTTGTACAAGAAAGCTGGGTTCAGAGCTCATCGTGGTGAA 3’ |
| *trim65* | 5’ GGGGACAAGTTTGTACAAAAAAGCAGGCTGCCACCATGGAGGATCACATGCAGTGC 3’ | 5’ GGGGACCACTTTGTACAAGAAAGCTGGGTTCATGGTCGCGGTTCACA 3’ |
